# Comparison of Materno-Neonatal Outcomes Between Cook Balloon and Prostaglandin Methods in Labor Induction Among Pregnancies Complicated by Fetal Growth Restriction: A Retrospective Cohort Study from France

**DOI:** 10.3390/jcm15166194

**Published:** 2026-08-10

**Authors:** Phuc Nhon Nguyen, Anna Ramos, Henri Marret

**Affiliations:** 1 Service de Gynécologie-Obstétrique, Maternité, Chirurgie Gynécologique, Centre Hospitalier Universitaire d’Orléans, 45100 Orléans, France; anna.ramos@chu-orleans.fr; 2 Faculté de Médecine, Université de Tours, 10 Bd Tonnellé, 37032 Tours, France; henri.marret@univ-tours.fr; 3 CHU Tours Bretonneau, 2 boulevard Tonnellé, 37044 Tours cedex 1, France

**Keywords:** Cook^®^ Balloon, cervical ripening, induced labor, prostaglandin, fetal growth restriction, vaginal birth

## Abstract

**Background**: To evaluate whether the methods of induction of labor (IOL) used for pregnancies complicated with fetal growth restriction (FGR) have different impacts on materno-fetal outcomes. **Methods**: This was a single-center, retrospective cohort study conducted over a one-year period, from January 2024 to December 2024, in the maternity ward of the Orléans University Hospital, France. The clinical data related to maternal–fetal characteristics and IOL outcomes were recorded. The results were comparable between the Cook^®^ balloon and Prostaglandin groups. **Results**: A total of 47 patients met the inclusion criteria; 27 cases underwent the Cook^®^ balloon and 20 cases received prostaglandin for IOL. There were no substantial differences between Cook balloon and prostaglandin on maternal–fetal characteristics and outcome of IOL. However, the duration of latent labor-delivery phase and duration of onset of labor induction and delivery were longer in the Cook^®^ balloon group compared with the prostaglandin group (30.05 ± 10.81 versus 22.29 ± 11.94 (hours) and 7.33 ± 3.96 versus 3.74 ± 4.77 (hours), respectively, *p* < 0.05). The success rate according to criterion 1 (Bishop score greater than 7 after IOL) was approximately 60% (28/47 cases). However, if using criterion 2 (success by vaginal birth), after the application of two or three methods of cervical ripening with amniotomy ± use of oxytocin, the success rate increased by more than 20%, up to 80.85% (38/47 cases). **Conclusions**: IOL with Cook^®^ balloon and prostaglandin may both be acceptable methods for pregnancies complicated by FGR. In addition, IOL did not differ in terms of materno-neonatal outcomes between two groups. However, the duration time from IOL to delivery and the duration from latent phase of labor to delivery were longer in the Cook^®^ balloon group compared with the prostaglandin group.

## 1. Introduction

Fetal growth restriction (FGR) is a common pregnancy complication worldwide, associated with an increased risk of stillbirth, neonatal morbidity and mortality, and long-term complications. It is defined as failure of the fetus to reach its full genetically determined growth potential due to pathological factors, primarily placental dysfunction. Diagnosis is based on ultrasound criteria established by the Delphi consensus, which distinguishes early FGR (<32 weeks of gestation) from late FGR (≥32 weeks of gestation) [1].

The management of FGR involves a strict monitoring of fetal growth and amniotic volume, as well as determining the optimal timing of delivery to balance the risks of stillbirth and prematurity [2]. In most cases, delivery is not anticipated before 37 weeks of amenorrhea, requiring induction of labor (IOL) [3]. Induction of labor aims to induce uterine contractions before the spontaneous onset of labor in order to promote vaginal delivery. It is indicated when the benefits of a planned delivery outweigh the risks associated with continuing the pregnancy [4,5,6]. Different methods can be used depending on national guidelines, local protocols, and the patient’s clinical characteristics. These methods include mechanical approaches, such as the Cook^®^ balloon, and pharmacological approaches using prostaglandin E1, prostaglandin E2, and oxytocin [7,8,9]. In a study conducted by Blanc-Petitjean et al., among 94 French maternity hospitals, 87 (92.6%) units reported using dinoprostone as a vaginal slow-released insert, 59 units used dinosprostone as a vaginal gel (62.8%) and 46 units used a balloon catheter (48.9%) for IOL procedure. Three maternity units reported the use of vaginal misoprostol [10].

In patients with FGR from 36 weeks of amenorrhea, IOL is a reasonable option, with the cesarean delivery rate being similar to that observed after spontaneous labor and neonatal outcomes being generally comparable [11]. In cases of severe small-for-gestational age (SGA) before 37 weeks of amenorrhea, labor induction allows for vaginal delivery in approximately two-thirds of cases. However, the results of studies on different inductions remain variable. According to Chavakula et al. in India, 25 µg vaginal misoprostol was more effective than Foley catheter for IOL in FGR [12]. In contrast, Villalain et al. in Spain reported that the use of Foley catheter was associated with a higher rate of vaginal delivery compared to dinoprostone and safety in both groups [13]. Moreover, as yet, there is a lack of studies evaluating the success of IOL using mechanical methods and pharmacological methods based on the Bishop score variability criteria and vaginal birth. These disparities warrant studies on different populations [5].

Thus, the objective of this study was to evaluate the maternal and neonatal outcomes of pregnancies complicated by late FGR undergoing IOL with the Cook^®^ balloon compared with prostaglandins at Orléans University Hospital.

## 2. Materials and Methods

### 2.1. Study Design

**Figure 1:**
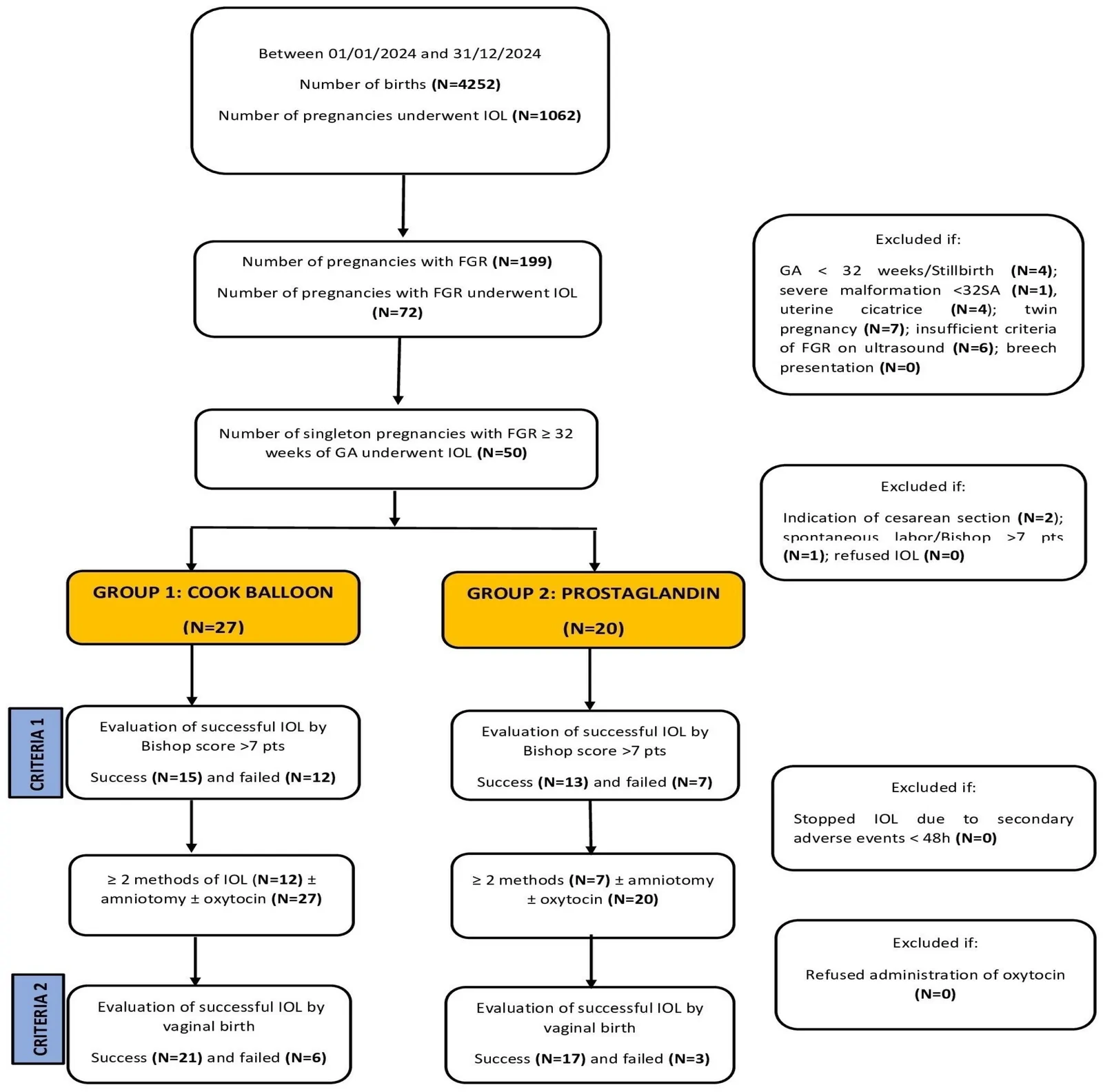
Study flowchart in the present study.

This was a single-center, retrospective cohort study. This study analyzed the records of pregnant women with fetal growth restriction (FGR) who underwent induction of labor (IOL) and gave birth between 1 January 2024 and 31 December 2024 in the maternity department of Orléans University’s Hospital, France (Figure 1). This study was accepted by the Clinical Research Room at the institution with the ethical code: 2025-HORS-RIPH-01.

Inclusion criteria included pregnancies diagnosed with FGR from 32 weeks of amenorrhea. These pregnant women had an unfavorable cervix (Bishop score ≤ 6) and underwent inpatient-setting IOL. The diagnosis of late FGR was made from 32 weeks of amenorrhea, in case of abdominal circumference or estimated fetal weight below the third percentile, or when at least two of the following three criteria were present: (1) abdominal circumference or an estimate of fetal weight below the 10th percentile; (2) decrease of two quartiles for abdominal circumference or fetal weight compared to previous measurements; (3) abnormal Doppler, defined by an umbilical artery pulsatility index above the 95th percentile, or a cerebroplacental ratio below the 5th percentile (mean) [11,14].

Non-inclusion criteria included twin pregnancy, breech presentation, scarred uterus, fetal death in utero, cesarean section before labor, fetus with morphological anomalies, IOL using combined methods of cervical ripening at the same time.

Pregnant women were divided into two groups depending on the IOL methods: the mechanical method (Cook^®^ balloon) and prostaglandin (PGE) method (Propess^®^, Angusta^®^). In cases where the patient was induced using two or three methods, the first method of IOL was used for the criterion 1 assessment. The IOL methods were based on patient’s preference after consideration of medical indication and consultation.

### 2.2. Data Collection

The variables were classified as continuous variables and categorical variables. The following variables were collected:

- General Variables: Maternal age (year); body mass index (BMI) (kg/m^2^); smoking; pregnancy pathologies, parity (time); medical history (hypertension, diabetes); obstetric comorbidities (gestational diabetes, preeclampsia, and others).
- FGR-Related Variables: Etiologies of FGR; estimated fetal weight (gram) following last ultrasound examination before induction; fetal weight (percentile) according to the growth chart; amount of amniotic fluid; and fetal Doppler indices.
- Obstetric Variables: Gestational age (weeks) at the diagnosis of FGR and at induction of labor; initial Bishop score.
- Neonatal Variables: Birth weight; APGAR score at 1 and 5 min; pH of umbilical artery; newborn sex; transfer to the neonatal intensive care unit (NICU) admission, and reasons.
- Induction-Related Variables: Bishop score at admission (points); IOL methods and number of methods.
- Complemental Variables: Gestational age at maturation (weeks); duration of cervical maturation (hours); presence of FHR abnormalities; FGR severity and diagnostic criteria (percentile, etiology, umbilical Doppler, amount of amniotic fluid); details of the course of maturation (complications, need for further maturation or artificial rupture of membranes, use of Syntocinon^®^) rupture of membranes, delivery method (vaginal birth, instrumental delivery, cesarean delivery); cesarean indications; reasons for instrumental delivery or cesarean; estimated blood loss (mL) during delivery; postpartum hemorrhage; degree of perineal tears; and postpartum infections.

### 2.3. Statistical Analysis

Data entry and analysis were imported and processed in SPSS version 27.0 (IBM, Armonk, NY, USA). The normality of raw data was determined by the Kolmogorov–Smirnov test and the Shapiro–Wilk test. Quantitative variables were described by their median and interquartile range (IQR) or mean and standard deviation (SD) according to normal distribution of data. Qualitative variables were presented as counts and percentages for each item.

The characteristics of the study population were compared between two groups (Cook^®^ balloon vs. prostaglandin). A comparison of maternal, obstetric, and neonatal characteristics between the two methods of labor induction was made using Student *t*-test for continuous variables, Chi-square test for categorical variables, and Fisher’s exact test for counts < 5.

The endpoints were compared between the “successful” and “unsuccessful” induction groups using a 2 × 2 table. The magnitude of the differences was measured using the odds ratio (OR) and 95% CI for binary variables. The significance threshold was set at 5% (*p* < 0.05).

## 3. Results

Over the period from 1 January 2024 to 31 December 2024, our center recorded 4252 births. In total, the number of pregnancies that underwent induction of labor (IOL) at our center was 1062 (24.97%). Our study found 199 FGR pregnancies (4.68%). Among them, 72 cases (36.18%) were induced. Among this population, 50 singleton fetuses with cephalic presentation beyond 32 weeks were recorded. Finally, our study found 47 eligible cases with the study criteria after the exclusion of 2 cases with an indication for cesarean section and 1 case of spontaneous labor. Among them, 27 cases underwent IOL using the Cook^®^ balloon and 20 cases used prostaglandin as the first method of IOL (Figure 1).

According to the results in Table 1, maternal characteristics were not significantly different between two IOL groups. The mean age was 28.96 ± 4.98 years, and 80.9% (38/47 cases) of the study population was between 20 and 34 years old. BMI before pregnancy was 23.14 kg/m^2^ and BMI during pregnancy was 27.00 kg/m^2^. In addition, fetal characteristics were comparable in both groups. The data shows a mean fetal weight equal to 2082.40 ± 297.38 g with a maximum at 2760 g and a minimum at 1416 g. In this sample, we noted that 25/47 (53.2%) of cases had EPF between 2000 and 2499 g. In general, the median percentile according to the last ultrasound was the third percentile.

The initial Bishop’s score (points) and the duration of labor induction (hours) were not substantially different between the Cook^®^ balloon and prostaglandin. However, the present study found that there was also a significant difference in the time from the onset of cervical ripening to delivery in hours (30.05 ± 10.81 vs. 22.29 ± 11.94 h) and the duration of latent labor at delivery (7.33 ± 3.96 vs. 3.74 ± 4.77 h) (Supplementary Figure S1). Indeed, prostaglandin had a duration between the onset of cervical ripening and delivery of less than 24 h (71.4%), while the Cook^®^ balloon was less common (28.6%). In addition, the rate of oxytocin use was higher in the mechanical group, comparable to the prostaglandin group (73.9% vs. 26.1%, *p* < 0.05) (Table 2).

**Figure 2:**
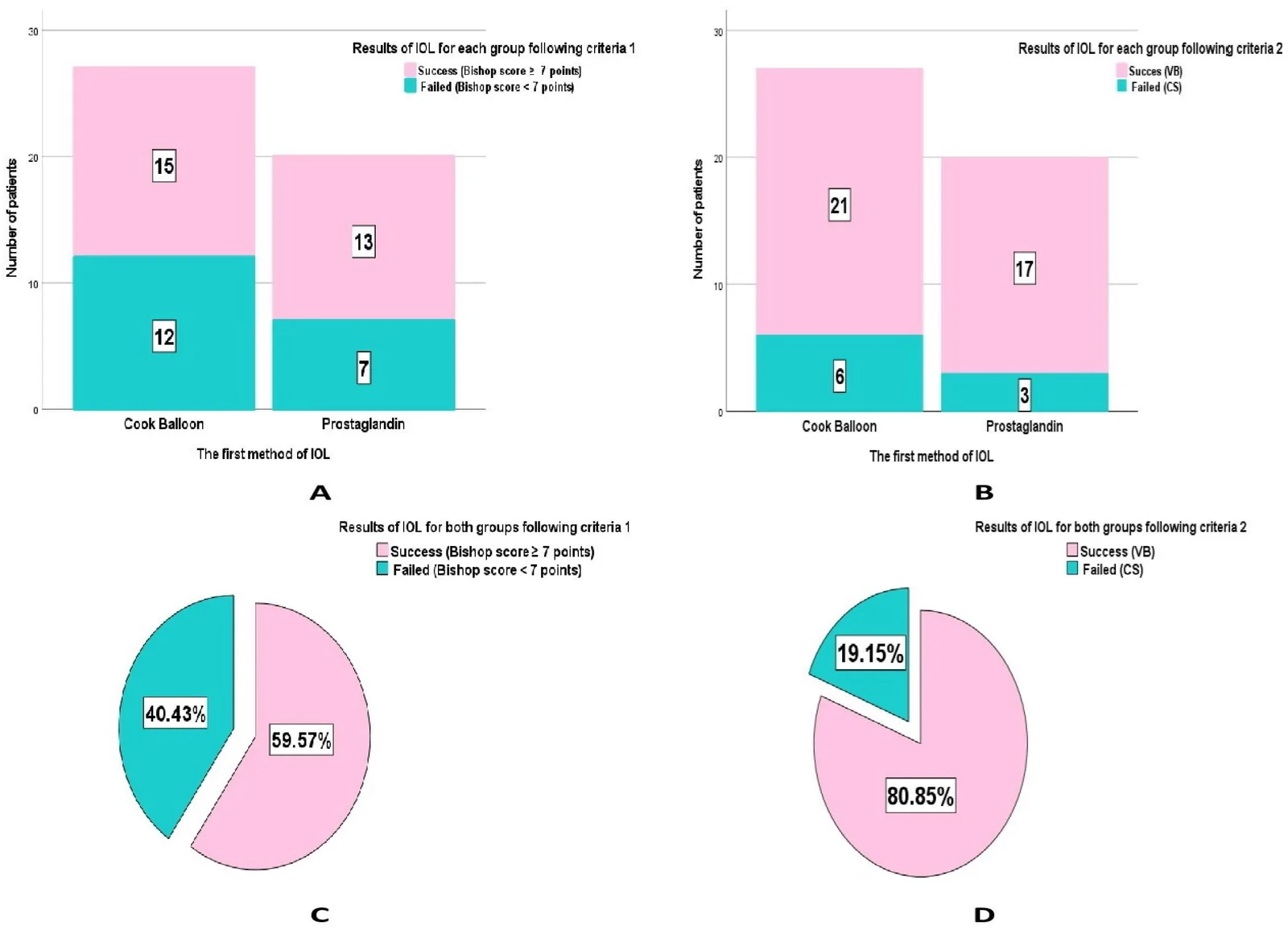
The result of IOL following two criteria between the two methods presents as frequency (n) following criterion 1–2 (**A**,**B**) and as percentage (%) following criterion 1–2 (**C**,**D**), respectively.

Figure 2 shows 15/27 (55.56%) successful cases in the Cook^®^ balloon group and 13/20 successful cases (65.0%) in the PGE group. On the other hand, according to criterion 2, the number of successful cases increased to 21/27 cases (77.78%) and 17/20 (85.0%), respectively. In general, the success rate using the Bishop score evaluation was greater than 7 after IOL according to criterion 1 (assessment according to the first method) was almost 60% (28/47 cases). However, if using criterion 2 (success by vaginal birth), after the application of two or three methods of cervical ripening with amniotomy ± use of oxytocin, the success rate increased by more than 20%, up to 80.85% (38/47 cases).

In our findings, there were no serious maternal side effects or an increase in cesarean section rate between the two groups. Overall, comparable rates of spontaneous and instrumental vaginal deliveries were observed in both groups (*p* > 0.05). However, there was a trend towards more cases with blood loss ≥ 500 mL and manual uterine revision rate in the Cook^®^ balloon group than in the prostaglandin group, but not significantly (*p* > 0.05). Overall, there were no great differences between the two groups of labor induction in neonatal outcomes (Apgar score, umbilical artery pH, and hospitalization in the neonatal unit). The mean arterial pH was 7.24 ± 0.11, and 14.89% (7/47) of cases were noted as having a pH less than 7.15. There were 12/47 cases (25.53%) hospitalized in the neonatal unit for low birth weight/respiratory distress (n = 5), morphological malformation (n = 4), and other reasons (n = 2) (Table 3).

In this study, according to the type of PGE, there were 4 cases using Propess^®^ tape, 20 cases using Angusta^®^ and 4 cases using both drugs after failure of one drug. The present study found no great differences related to materno-neonatal outcomes were observed among pharmacological groups, except for IOL duration time (Supplementary Table S1).

## 4. Discussion

In this study, the rate pregnancies complicated by fetal growth restriction (FGR) that underwent induction of labor (IOL) was 36.18%. This rate was similar to the study of Maria J. Rodriguez-Sibaja (32%) [15]. In the present study, if the study evaluated the success rate as a Bishop score greater than seven points after the first method of induction of labor (IOL), the failure rate (Bishop score less than 7) was up to 40.43%. However, if the study evaluated the success rate as vaginal delivery, the failure rate (cesarean delivery) reduced to 19.15%. Our cesarean rate is lower than that of other studies. In the study of Pinton et al., of the 146 women included, 56 cases (38.4%) underwent cesarean delivery (CD) [16]. In the study of Metrop et al., among 320 patients, 246 cases delivered vaginally (76.9%) and 74 cases underwent CD (23.1%) [17].

Our low rate of CD may be explained by our prompt management in IOL. In fact, we combined two or three methods of IOL, including balloon catheter, dinosprostone, and misoprostol, in cases where the first method failed. In addition, the IOL was performed with the patient’s consent. In France, most maternity units used two (39.4%) or three or more (35.1%) agents for cervical ripening [10]. In general, IOL for FGR was not associated with an increased rate of CD. In the study of Kehl et al., 120 pregnancies in the FGR group and 2210 pregnancies in the control group underwent IOL; the CD rate was not different between the two groups (27.0 vs. 26.2%, *p*  =  0.9154) [18].

According to Chavakula et al., women induced with misoprostol were less likely to deliver by lower-segment cesarean delivery (15.2% versus 29.6%; *p* = 0.168) and to require oxytocin augmentation (60.9% versus 85.2%; *p* = 0.007) [12]. Similarly, among 266 women, 131 (49.2%) underwent induction by PGE_2_, 116 (43.6%) by EAB, and 19 (7.14%) by both methods. The rate of CD (17.24% vs. 6.11% vs. 10.53%, *p* =  0.022) and maternal composite outcome (18.97% vs. 6.11% vs. 10.53%, *p* <  0.01) was higher among women who underwent induction using extra-amniotic balloon (EAB) compared with PGE_2_ or both [19].

However, in the study of Al-Hafez et al. [20], the rate of CD was higher in the inductions that received prostaglandin than those that did not (25.5% vs. 14.8%, adjusted odds ratio, 1.80; 1.07–3.02). The rate of CD for non-reassuring fetal heart tracings was higher for those that received prostaglandin than those did not (16.0% vs. 8.7%, adjusted odds ratio, (aOR): 2.37; 95% confidence interval (CI): 1.28–4.41) [20]. Similar to the study of Di Mascio et al., in pregnancies complicated by late FGR, IOL with mechanical methods is associated with a lower risk of uterine tachysystole, cesarean section or operative delivery for non-reassuring fetal status, and adverse neonatal outcomes compared with pharmacological methods. A total of 571 pregnancies complicated by late FGR who underwent IOL (391 pregnant women with dinoprostone and 180 pregnant women with mechanical methods) were included in the analysis. The incidence of uterine tachysystole (19.2% vs. 5.6%; *p* = 0.001) was higher in women undergoing IOL with dinoprostone than in those undergoing IOL with mechanical methods. Similarly, the incidence of CD or operative delivery for non-reassuring fetal status was 25.6% vs. 17.2%; *p* = 0.027 [21]. In accordance with Gimovsky et al., the incidence of CD for “non-reassuring” heart rate was 18.1% in the misoprostol group (13/72), 13.6% in the dinoprostone group (9/66) and 6.5% in the mechanical/oxytocin group (32/494), *p* = 0.001. Overall incidence of CD was 31.9% (23/72) with misoprostol, 18.2% (12/66) with dinoprostone and 11.1% (55/494) with mechanical/oxytocin induction (*p* < 0.001). The study concluded that IOL using misoprostol resulted in the highest incidence of CD for “non-reassuring” fetal heart rate in comparison to mechanical methods [22]. In the general population of IOL at term, meta-analysis confirmed a comparable CD rate (20% versus 22%, risk ratio (RR): 0.90, 95% CI: 0.54–1.50) and showed fewer cases of hyperstimulation when a Foley catheter was used [23]. Compared with vaginal dinoprostone, composite adverse maternal outcomes were significantly lower with low-dose vaginal misoprostol (adjusted odds ratio (aOR): 0.80, 95% CI: 0.65–0.98, *p*  =  0.03, *I*^2^  =  0%) [24].

Regarding neonatal outcomes, our study recorded 12 newborns (25.53%) admitted to NICU due to malformations (n = 4), SGA/respiratory distress (n = 5), and neonatal jaundice, and PROM > 18 h. Four cases recorded an Apgar score less than seven points at 5 min. This study found no significant differences in terms of neonatal outcomes between the Cook^®^ Balloon and prostaglandin groups. In general, exposure to prostaglandin in pregnant women undergoing induction of labor and delivering term SGA neonates was not associated with an increased risk for CD for abnormal CTG, cesarean for any reason, or neonatal morbidity [25]. However, compared to pregnancies without FGR, in the FGR group, abnormal CTG was more common (30.8 vs. 21.9%, *p*  =  0.0214), and fetal blood analysis was required more often (2.5 vs. 0.5%, *p*  =  0.0261). There were more postpartum transfers to the NICU in the FGR group (40.0 vs. 12.8%, *p*  <  0.0001) [18]. Of 756 (0.3%) inductions, 212 (28%) used prostaglandin (108 prostaglandin E1, 94 prostaglandin E2), and 553 (72%) used non-prostaglandin methods, including oxytocin (348, 63%), amniotomy (211, 38%), and/or mechanical dilation (9, 1%). There were no differences in the composite of adverse neonatal outcomes between the prostaglandin (10.4%) and the non-prostaglandin group (6.7%); adjusted odds ratio, 1.39 (0.64–3.03). However, when Prostaglandin E1 and Prostaglandin E2 were examined independently, there were similar increases in the composite of adverse neonatal outcomes and CD rates for both Prostaglandin E1 and Prostaglandin E2 compared with non-prostaglandin controls [20].

Accordingly, composite adverse neonatal outcome (26.1% vs. 16.7%; *p* = 0.013) and NICU admission (16.9% vs. 5.6%; *p* < 0.001) was higher in women undergoing IOL with dinoprostone than in those undergoing IOL with mechanical methods [21]. Similarly, Gimovsky et al. revealed that newborn resuscitation was more common in the dinoprostone group compared to mechanical group, but showed no increase compared to misoprostol. Resuscitation occurred in 2.8%, 4.6%, and 0.8% of the misoprostol, dinoprostone, and mechanical/oxytocin groups, respectively (*p* = 0.03) [22]. Nevertheless, our study found no differences in neonatal outcomes between the two groups. This may be due to the small sample size in the present study. In general, our study reported no serious complications relating to maternal or neonatal morbidity. This finding was similar to other studies [23]. Furthermore, in our study, normal Doppler ultrasound scan and gestational age at IOL greater than 37 weeks showed good prognostic factors for vaginal delivery [26].

### Strengths and Limitations

The study explores a clinically relevant topic and provides preliminary data on induction strategies in pregnancies affected by fetal growth restriction at our tertiary referral hospital. The major maternal and pregnancy clinical characteristics potentially affecting the primary outcome were similar among groups and were accounted for in the statistical analysis. Our study was conducted at the consultant hospital with standardized guidelines. Thus, the ultrasound diagnosis of FGR was reliable and the clinical management was strictly monitored. The hospital protocol applied all methods of IOL and combinations of two or three methods without adverse materno-neonatal outcomes. Other centers used only one method of IOL and evaluated the result of IOL after 24 h. Therefore, this study contributes important data with practical application. However, the sample size was small and the retrospective design could inevitably limit statistical power, particularly for neonatal outcomes and uncommon maternal complications. The outcomes were subject to intervention bias including an evaluation of IOL results and decision-making time. Further randomized-controlled trial studies with large number of patients are required [5,27].

## 5. Conclusions

Both Cook^®^ balloon and prostaglandin are likely safe and effective methods for the induction of labor among pregnancies complicated by FGR. Nevertheless, the duration from IOL to delivery and the duration from latent phase of labor to delivery were longer in the mechanical group compared with the PGE group. The success rate of IOL increased after using ≥2 methods of cervical ripening with amniotomy ± use of oxytocin.

Moreover, the IOL methods should be carefully applied based on medical indication and patient’s choice. Further evidence is required to obtain findings in different populations.

## Figures and Tables

**Table 1:** Materno-fetal characteristics in terms of induction of labor methods.

Characteristics		Overall (N = 47)	Cook Balloon (N = 27)	Prostaglandin (N = 20)	*p*-Value
Maternal age (years)	Mean ± SD (min–max)	28.96 ± 4.98 (19–40)	29.52 ± 4.64 (20–38)	28.20 ± 5.44 (19–40)	0.375 ^a^
Pre-pregnancy BMI (kg/m^2^)	Median [IQR: 25–75%] (min–max)	23.14 [20.45–28.08] (15.57–43.82)	21.48 [19.47–28.00] (15.57–43.82)	25.36 [22.19–29.88] (18.82–37.02)	0.060 ^b^
BMI in pregnancy (kg/m^2^)	Median [IQR: 25–75%] (min–max)	27.00 [23.18–31.65] (20.00–44.64)	25.48 [23.00–29.66] (20.00–44.64)	29.19 [25.66–32.87] (20.30–37.90)	0.102 ^b^
Smoking	No	34 (100.0)	21 (61.8)	13 (38.2)	0.511 ^c^
	Yes	13 (100.0)	6 (46.2)	7 (53.8)	
Gravida	Mean ± SD (min–max)	1.23 ± 1.45 (0–7)	1.26 ± 1.63 (0–7)	1.20 ± 1.20 (0–4)	0.891 ^d^
	≥1	28 (100.0)	15 (53.6)	13 (46.4)	0.561 ^c^
	0	19 (100.0)	12 (63.2)	7 (36.8)	
Parity	Mean ± SD (min–max)	0.57 ± 0.88 (0–3)	0.44 ± 0.85 (0–3)	0.75 ± 0.91 (0–3)	0.243 ^a^
Obstetrical history	Stillbirth > 22 weeks of GA	0 (0.0)	0 (0.0)	0 (0.0)	NA
	FGR	0 (0.0)	0 (0.0)	0 (0.0)	NA
Pathologies in pregnancy	No	32 (100.0)	19 (49.4)	13 (40.6)	0.716 ^d^
	Gestational hypertension disorders	1 (100.0)	1 (100.0)	0 (0.0)	
	Pre-eclampsia	6 (100.0)	3 (50.0)	3 (50.0)	
	Non-insulin-dependent pregnant diabetics	6 (100.0)	3 (50.0)	3 (50.0)	
	Insulin-dependent diabetes mellitus	1 (100.0)	0 (0.0)	1 (100.0)	
	Others *	4 (100.0)	3 (75.0)	1 (25.0)	
Rhesus	Positive	41 (100.0)	23 (56.1)	18 (43.9)	1.0 ^d^
	Negative	6 (100.0)	4 (66.7)	2 (33.3)	
Gestational age at detection (weeks)	Median [IQR: 25–75%] (min–max)	35.4 [31.5–36.4] (22.0–38.0)	35.0 [31.3–36.1] (23.0–37.4)	36.0 [33.2–36.5] (22.0–38.0)	0.232 ^b^
Amniocentesis	Yes	2 (100.0)	2 (100.0)	0 (0.0)	0.500 ^d^
	No	45 (100.0)	25 (55.6)	20 (44.4)	
Etiologies of FGR	Unknown	35 (100.0)	20 (57.1)	15 (42.9)	1.0 ^d^
	Vascular	8 (100.0)	4 (50.0)	4 (50.0)	
	Infection	2 (100.0)	1 (50.0)	1 (50.0)	
	Chromosomal	1 (100.0)	1 (100.0)	0 (0.0)	
	Vascular et infection	1 (100.0)	1 (100.0)	0 (0.0)	
EFW following the last US (gram)	Mean ± SD (min–max)	2082.40 ± 297.38 (1416–2760)	2047.41 ± 298.68 (1416–2705)	2129.65 ± 296.55 (1498–2760)	0.354 ^a^
	≤1999	18 (100.0)	13 (72.2)	5 (27.8)	0.273 ^d^
	2000–2499	25 (100.0)	12 (48.0)	13 (52.0)	
	≥2500	4 (100.0)	2 (50.0)	2 (50.0)	
EFW following the last US (percentile)	Median [IQR: 25–75%] (min-max)	3 [1–8] (0–10)	3 [0–7] (0–9)	5 [2–9] (0–10)	0.104 ^b^
	<3	20 (100.0)	12 (60.0)	8 (40.0)	0.803 ^c^
	3–5	11 (100.0)	7 (63.6)	4 (36.4)	
	>5	16 (100.0)	8 (50.0)	8 (50.0)	
Break in the utero growth curve	Present	39 (100.0)	20 (51.3)	19 (48.7)	0.114 ^d^
	Absent	8 (100.0)	7 (87.5)	1 (12.5)	
Gestational age at IOL (weeks)	Mean ± SD (min-max)	37.6 ± 1.3 (34.5–40.3)	37.4 ± 1.3 (34.5–40.3)	38.0 ± 1.2 (35.6–40.1)	0.133 ^a^

Data was presented as n (%), mean ± SD (min–max) and median [IQR: 25–75%]. Prostaglandin including dinoprostone (Propess^®^) and misoprostone (Angusta^®^), and a combination of two PGE methods. ^a^ Independent sample test; ^b^ independent-sample Mann–Whitney U test; ^c^ Chi-square test; ^d^ Fisher exact test. * Others: systemic lupus erythematosus, gestational cholestasis. BMI: body mass index (kg/m^2^); EFW: estimated fetal weight; GA: gestational age; NA: not applicable; FGR: fetal growth restriction; US: ultrasound.

**Table 2:** Characteristics of the study population between two methods of induction of labor.

Characteristics		Overall (N = 47)	Cook Balloon (N = 27)	Prostaglandin (N = 20)	*p*-Value
Duration time between IOL and delivery (hours) *	Mean ± SD (min–max)	26.58 ± 11.84 (9.5–54.5)	30.05 ± 10.81 (15.0–54.5)	22.29 ± 11.94 (9.5–46.5)	0.043 ^a^
	<24	14 (100.0)	4 (28.6)	10 (71.4)	0.018 ^b^
	≥24	24 (100.0)	17 (70.8)	7 (29.2)	
Duration time between latent phase of labor and delivery (hours) *	Mean ± SD (min–max)	5.72 ± 4.64 (0.5–19.0)	7.33 ± 3.96 (0.5–16)	3.74 ± 4.77 (0.5–19.0)	0.015 ^a^
	<12	36 (100.0)	19 (52.8)	17 (47.2)	0.492 ^c^
	≥12	2 (100.0)	2 (100.0)	0 (100.0)	
Bishop score before IOL (points)	Mean ± SD (min–max)	2.89 ± 1.82 (0–6)	2.52 ± 1.85 (0–6)	3.40 ± 1.70 (0–5)	0.101 ^d^
Bishop score at IOL evaluation	Mean ± SD (min–max)	6.28 ± 2.72 (1–12)	5.96 ± 2.58 (1–10)	1.70 ± 2.92 (2–12)	0.365 ^d^
Variability of Bishop score (at evaluation—before IOL)	Mean ± SD (min–max)	3.40 ± 2.23 (0–8)	3.48 ± 2.33 (0–8)	3.30 ± 2.16 (0–7)	0.786 ^d^
	≥3	31 (100.0)	17 (54.8)	14 (45.2)	0.758 ^b^
	<3	16 (100.0)	10 (62.5)	6 (37.5)	
Result of IOL based on Bishop score (Criteria 1)	Succes (Bishop ≥ 7 pts)	28 (100.0)	15 (53.6)	13 (46.4)	0.561 ^b^
	Failed (Bishop < 7 pts)	19 (100.0)	12 (63.2)	7 (36.8)	
Result of IOL based on mode of delivery (Criteria 2)	Success (vaginal delivery)	38 (100.0)	21 (55.3)	17 (44.7)	0.713 ^c^
	Failed (cesarean section)	9 (100.0)	6 (66.7)	3 (33.3)	
Number of IOL methods	1	34 (100.0)	18 (52.9)	16 (47.1)	0.348 ^b^
	≥2	13 (100.0)	9 (69.2)	4 (30.8)	
Uterine hyperstimulation and abnormal CTG during IOL	Absent	40 (100.0)	22 (55.0)	18 (45.0)	0.351 ^c^
	Uterine hypertonia with CTG abnormalities	1 (100.0)	1 (100.0)	0 (0.0)	
	Uterine tachysystole with CTG abnormalities	1 (100.0)	0 (0.0)	1 (100.0)	
	Isolated CTG abnormalities	5 (100.0)	4 (80.0)	1 (20.0)	
Undesirable events	Present	0 (0.0)	0 (0.0)	0 (0.0)	NA
	Absent	47 (100.0)	27 (57.4)	20 (42.6)	
Spontaneous rupture of amniotic fluid membrane (%)	Present	14 (100.0)	7 (50.0)	7 (50.0)	0.535 ^b^
	Absent	23 (100.0)	20 (60.6)	13 (39.4)	
Color of amniotic fluid	Clear	44 (100.0)	26 (59.1)	18 (40.9)	0.567 ^c^
	Meconium	3 (100.0)	1 (33.3)	2 (66.7)	
Maximum dose of oxytocin (mUI/min)	Median [IQR: 25–75%] (min–max)	4.0 [4.0–6.0] (2.0–12.0)	4.0 [4.0–6.0) (2.0–10.0)	4.4 [4.0–9.0] (4.0–12.0)	0.467 ^d^
Pushing time in delivery (mins)	Median [IQR: 25–75%] (min–max)	9.5 [4.0–17.5] (1–44)	12.0 [5.5–19.0] (1–44)	4 [2.0–11.0] (1–26)	0.066 ^d^
Requirement of peridural anesthesia	Yes	34 (100.0)	22 (64.7)	12 (35.3)	0.186 ^b^
	No	13 (100.0)	5 (38.5)	8 (61.5)	

^a^ Independent sample test; ^b^ Chi-square test; ^c^ Fisher exact test; ^d^ independent-sample Mann–Whitney U Test. * Total N = 38 including Cook^®^ balloon (n = 21) and prostaglandin (n = 17). CTG: cardiotocography; IOL: induction of labor; IQR: inter-quartile range. Undesirable events of Cook^®^ balloon including drop out of balloon after insertion or requiring withdrawal of Cook^®^ balloon before the time-point of evaluation. Undesirable events of PGE include dropped Propess^®^, vomiting or allergic reaction. Number cases of hemorrhage (n = 0) and fever (n = 0).

**Table 3:** Materno-neonatal outcomes following the methods of induction of labor.

Variables	Characteristic	Overall (N = 47)	Cook Balloon (N = 27)	Prostaglandin (N = 20)	*p*-Value
Vaginal birth	Spontaneous	33 (100.0)	17 (51.5)	16 (48.5)	0.609 ^a^
	Assisted instrument *	4 (100.0)	3 (75.0)	1 (25.0)	
Indication of CS	Failed IOL	2 (100.0)	1 (50.0)	1 (50.0)	NA
	Non engagement	0 (0.0)	0 (0.0)	0 (0.0)	
	Abnormal fetal heart rate pattern before 4 cm dilation	6 (100.0)	5 (83.3)	1 (16.7)	
	Abnormal fetal heart rate pattern in labor	0 (0.0)	0 (0.0)	0 (0.0)	
	Bradycardia	0 (0.0)	0 (0.0)	0 (0.0)	
	Stagnation of dilatation	0 (0.0)	0 (0.0)	0 (0.0)	
Estimated blood loss (mL)	Median [IQR: 25–75%] (min–max)	200 [100–350] (50–1000)	200 [100–600] (50–1000)	150 [100–287.50] (50–600)	0.384 ^b^
	<500	38 (100.0)	19 (50.0)	19 (50.0)	0.059 ^a^
	≥500	9 (100.0)	8 (88.9)	1 (11.9)	
Postpartum hemorrhage	Present **	6 (100.0)	5 (83.3)	1 (16.7)	0.221 ^a^
	Absent	41 (100.0)	22 (53.7)	19 (46.3)	
Perineal status	Intact	13 (100.0)	8 (61.5)	5 (38.5)	NA
	Minor tears	13 (100.0)	7 (53.8)	6 (46.2)	
	First-degree laceration	14 (100.0)	8 (57.1)	6 (42.9)	
	Second-degree laceration	2 (100.0)	1 (50.0)	1 (50.0)	
	Third-degree laceration	0 (0.0)	0 (0.0)	0 (0.0)	
	Fourth-degree laceration	0 (0.0)	0 (0.0)	0 (0.0)	
Manual revision of uterine cavity	Yes	5 (100.0)	5 (100.0)	0 (0.0)	0.06 ^a^
	No	37 (100.0)	19 (51.4)	18 (48.6)	
Hemoglobin (g/dL)	Mean ± SD (min–max)	10.93 ± 1.63 (6.8–14.0)	10.74 ± 1.68 (6.8–14.0)	11.25 ± 1.25 (9.2–14.0)	0.371 ^c^
	<7	1 (100.0)	1 (100.0)	0 (0.0)	1.0 ^a^
	≥7	35 (100.0)	22 (62.9)	13 (37.1)	
Other complications ***	Present	4 (100.0)	3 (75.0)	1 (25.0)	0.626 ^a^
	Absent	43 (100.0)	24 (55.8)	19 (44.2)	
Length of hospital stay in postpartum course (days)	Mean ± SD (min–max)	4.15 ± 1.59 (2–10)	4.22 ± 1.70 (3–10)	4.05 ± 1.47 (2–10)	0.717 ^c^
	<4	33 (100.0)	19 (57.6)	14 (42.4)	1.0 ^a^
	≥4	14 (100.0)	8 (57.1)	6 (42.9)	
Newborn weight (gram)	Mean ± SD (min–max)	2311.79 ± 394.13 (1485–3305)	2260.19–461.75 (1485–3305)	2381.45–274.63 (1925–2870)	0.268 ^c^
Newborn sex	Female	25 (100.0)	11 (44.0)	14 (56.0)	0.076 ^d^
	Male	22 (100.0)	16 (72.7)	6 (27.3)	
Apgar score at 1 min (points)	Median [IQR: 25–75%] (min–max)	9 [6–10] (1–10)	8 [6–10] (1–10)	10 [6.3–10] (1–10)	0.205 ^b^
	<5	7 (100.0)	5 (71.4)	2 (28.6)	0.682 ^a^
	≥5	40 (100.0)	22 (55.0)	18 (45.0)	
Apgar score at 5 min (points)	Median [IQR: 25–75%] (min–max)	10 [10–10] (3–10)	10 [9–10] (3–10)	10 [10–10] (6–10)	0.338 ^b^
	<7	4 (100.0)	3 (75.0)	1 (25.0)	0.626 ^a^
	≥7	43 (100.0)	24 (55.8)	19 (44.2)	
pH of umbilical artery	Mean ± SD (min–max)	7.24 ± 0.11 (6.83–7.40)	7.23 ± 0.12 (6.83–7.40)	7.25 ± 0.09 (7.02–7.36)	0.383 ^c^
	pH < 7.15	7 (100.0)	5 (71.4)	2 (28.6)	0.446 ^a^
	pH ≥ 7.15	39 (100.0)	21 (53.8)	18 (46.2)	
NICU admission	Yes	12 (100.0)	9 (75.0)	3 (25.0)	0.191 ^d^
	No	35 (100.0)	18 (51.4)	17 (48.6)	
Reason for NICU admission	Malformations ^+^	5 (100.0)	3 (60.0)	2 (40.0)	1.0 ^a^
	SGA/respiratory distress	5 (100.0)	4 (80.0)	1 (20.0)	
	Others ^++^	2 (100.0)	2 (100.0)	0 (0.0)	
Length of hospital stay in NICU (days)	Mean ± SD (min–max)	4.75 ± 1.66 (2–7)	4.80 ± 1.69 (2–7)	4.50 ± 2.12 (3–6)	0.677 ^b^
	<7	9 (100.0)	6 (66.7)	3 (33.3)	0.509 ^a^
	≥7	3 (100.0)	3 (100.0)	0 (0.0)	

^a^ Fisher exact test; ^b^ independent-sample Mann–Whitney U test; ^c^ independent sample test ^d^ Chi-square test. * N = four spatulas; ** etiologies include atony (n = 2) and vaginal laceration (n = 4). *** Other complications: urinary infection, vaginitis; preeclampsia and gestational hypertension. No endometritis and no blood transfusion. ^+^ Malformation relating to heart, lung, and brain. ^++^ Others: neonatal jaundice, pre-labor rupture of membranes (PROM) > 18 h. NICU: neonatal intensive care unit; SGA: small for gestational age.

## Data Availability

The datasets generated and/or analyzed during the current study are not publicly available due to the restricted policy of the hospital but are available from the corresponding author on reasonable request.

## Supplementary Materials

The following supporting information can be downloaded at: https://www.mdpi.com/article/10.3390/jcm15166194/s1, Figure S1: Duration from the beginning of IOL to delivery following the first method of IOL; Table S1: Comparison of materno-neonatal characteristics using different types of prostaglandins.

## Abbreviations

CS	Cesarean section
CI	Confidence interval
CTG	Cardiotocography
IOL	Induction of labor
OR	Odds ratio
FGR	Fetal growth restriction
