# Supplementary material for: Comparison of Materno-Neonatal Outcomes Between Cook Balloon and Prostaglandin Methods in Labor Induction Among Pregnancies Complicated by Fetal Growth Restriction: A Retrospective Cohort Study from France
Source: J Clin Med. 2026 Aug 10;15(16):6194. doi: 10.3390/jcm15166194 (PMC13513655; doi:10.3390/jcm15166194)
Supplement: Supplementary file 1 [file jcm-15-06194-s001.zip › jcm-4398137-supplementary.pdf]

Supplementary Materials

Table S1. Comparison of materno-neonatal characteristics following the types of prostaglandins.

Variables	Characteristics	Propess (N = 4)	Angusta (20)	≥ 2 Methods of Prostaglandin (N = 4)	*p*-Value
IOL result (Criteria 1)	Success	3 (21.4)	11 (78.6)	0 (0.0)	0.109 [a]
	Failed	1 (7.1)	9 (64.3)	4 (28.6)	
IOL result (Criteria 2)	Vaginal birth	4 (16.0)	19 (76.0)	2 (8.0)	0.090 [a]
	Cesarian section	0 (0.0)	1 (33.3)	2 (66.7)	
Duration of IOL (hours)	Median [IQR: 25–75%] (min–max)	12.0 [10.5–19.5) (10.0–22.0)	22.5 [14.0–27.3] (5.5–47.0)	49.5 [28.0–72.5] (25.0–82.0)	0.006 [b]
Interval between latent phase of labor and delivery (hours) *	Mean ± SD (min–max)	6.13 ± 8.72 (0.5–19.0)	4.74 ± 4.31 (0.5–16.0)	1.50 ± 1.41 (0.5–2.5)	0.580 [c]
Interval between IOL and delivery (hours)*	Mean ± SD (min–max)	20.25 ± 13.99 (11.0–41.0)	27.58 ± 13.99 (9.5–54.5)	41.75 ± 6.72 (37.0–46.4)	0.218 [c]
Initial Bishop score (points)	Mean ± SD (min–max)	3.50 ± 1.29 (2–5)	3.40 ± 1.27 (1–5)	1.50 ± 2.38 (0–5)	0.069 [c]
Variability of Bishop score (points) (after-before IOL)	Mean ± SD (min–max)	4.50 ± 3.11 (0–7)	2.70 ± 1.78 (0–7)	1.75 ± 1.26 (0–3)	0.138 [c]
	≥3	3 (17.6)	13 (76.5)	1 (5.9)	0.463 [a]
	<3	1 (9.1)	7 (63.6)	3 (27.3)	
Estimated blood loss (ml)	Median [IQR: 25–75%] (min–max)	175.0 [112.5–500] (100–600)	175.0 [100–300] (50–650)	200 [112.5–287.5] (100–300)	0.877 [b]
	<500	3 (11.5)	19 (73.1)	4 (15.4)	0.497 [a]
	≥500	1 (50.0)	1 (50.0)	0 (0.0)	
pH of umbilical artery	Mean ± SD (min-max)	7.28 ± 0.69 (7.20–7.34)	7.25 ± 0.09 (7.07–7.37)	7.19 ± 0.12 (7.02–7.28)	0.379 [a]
	pH < 7.15	0 (0.0)	2 (66.7)	1 (33.3)	0.669 [c]
	pH ≥ 7.15	4 (16.7)	17 (70.8)	3 (12.5)	
Apgar score at 1 min (points)	Median [IQR: 25–75%] (min–max)	10.0 [7.75–10.0] (7–10)	9.5 [6.25–10.0] (1–10)	9.5 [6.75–10.0] (6–10)	0.678 [b]
	<5	0 (0.0)	2 (100.0)	0 (0.0)	1.0 [a]
	≥5	4	18	4	

		(15.4)	(69.2)	(15.4)	
Apgar score at 5 min (points)	Median [IQR: 25–75%] (min–max)	10 [10–10] (10–10)	10 [10–10] (6–10)	10.0 [10.0–10.0] (10.0–10.0)	0.406 [b]
	<7	0 (0.0)	1 (100.0)	0 (0.0)	1.0 [a]
	≥7	4 (14.8)	19 (70.4)	4 (14.8)	
NICU admission	Yes	0 (0.0)	4 (100.0)	0 (0.0)	1.0 [a]
	No	4 (16.7)	16 (66.7)	4 (16.7)	
Length of hospital stay in NICU (days)	<7	0 (0.0)	3 (100.0)	0 (0.0)	NA
	≥7	0 (0.0)	1 (100.0)	0 (0.0)	

[a] Fisher exact test; [b] Kruskal Wallis test (Asymp. Sig.); [c] One-way Anova test * Propess (n = 4); Angusta (n = 19); and combination of 2 methods of PGE (n = 2). IOL: Induction of labor.

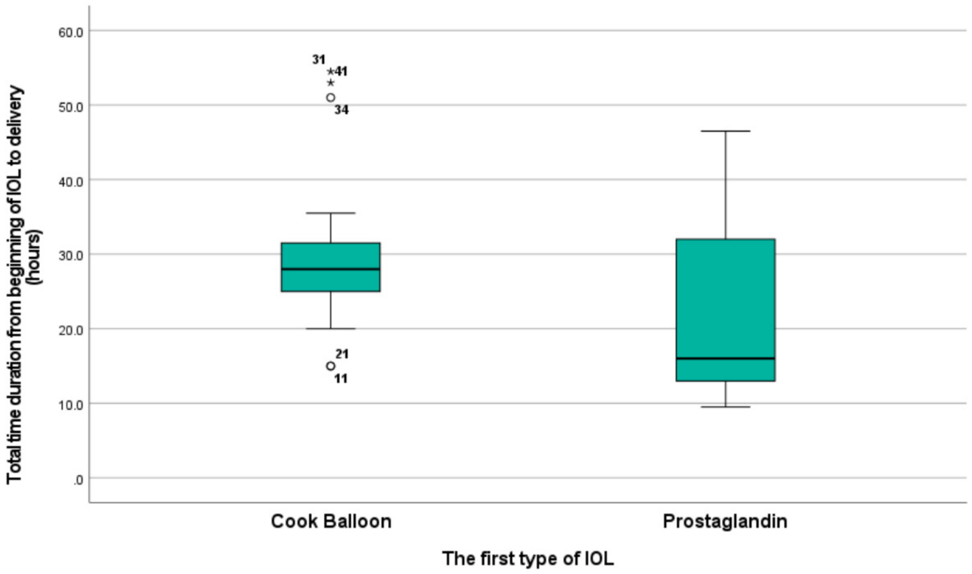

Figure S1. Duration time from the beginning of IOL to delivery following the first method of IOL.

The circles and asterisks in Supplementary Figure S1 are the order number of patients who have the outliers of total duration time from beginning of IOL to delivery in the Cook balloon group. The number 11 and 21 present under the median value of a box plot corresponding to 15.0 (hours). The number 31, 34, and 41 present above the median value of a box plot corresponding to 54.5 (hours), 51.0 (hours), and 53.0 (hours), respectively.
